# Mechanical and Sorptivity Characteristics of Edge-Oxidized Graphene Oxide (EOGO)-Cement Composites: Dry- and Wet-Mix Design Methods

**DOI:** 10.3390/nano8090718

**Published:** 2018-09-12

**Authors:** Yousef Alharbi, Jinwoo An, Byoung Hooi Cho, Mohammad Khawaji, Wonseok Chung, Boo Hyun Nam

**Affiliations:** 1 Department of Civil, Environmental, and Construction Engineering, University of Central Florida, 4000 Central Florida Blvd, Orlando, FL 32816, USA; yrharbi@knights.ucf.edu (Y.A.); jinwooan@knights.ucf.edu (J.A.); byoungcho@ucf.edu (B.H.C.); mkhawaji@knights.ucf.edu (M.K.); 2 Department of Civil Engineering, Kyung Hee University, 1732 Deogyeong-daero, Giheung-gu, Yongin-si, Gyeonggi-do 17104, Korea

**Keywords:** graphene oxide, mix design, cement composites, porosity, water sorptivity, mechanical properties

## Abstract

This paper aims to investigate the effects of edge-oxidized graphene oxide nanoflakes (EOGO) on the mechanical properties and sorptivity of cement composites. The EOGO used in this study was produced by a mechanochemical process that assists the production of EOGO in large quantities at significantly reduced costs, enabling its practical use for infrastructure construction. The scope of this work includes the use of EOGO as an additive in cement composites, including cement paste and mortar. This study explores two mixing methods: The dry-mix method and the wet-mix method. The dry-mix method uses EOGO as dry powder in cement composites whereas the wet-mix method uses a water-dispersed solution (using a sonication process). Varied percentages of EOGO, ranging from 0.01% to 1.0%, were used for both methods. To evaluate the concrete durability, the effect of EOGO addition on sorptivity of the cement composites was investigated by performing total porosity and water sorptivity tests. It was found that 0.05% of EOGO is the optimum proportion to exert the highest strength in compressive and flexural strength tests. In addition, the dry-mix method is comparable to the wet-mix method (with dispersion of EOGO), thus more practical for field engineering applications.

## 1. Introduction

The strength and durability of concrete are the two most significant characteristics that govern structural efficiency for load capacity and service life purposes [1]. Strength is a function of different aspects such as mix design, structural design, and the curing process [1]. Durability of concrete, which controls the concrete’s service life, may be defined as the capability to maintain a minimum performance level over a specific time when exposed to a degrading environment [2]. Concrete durability has been covered by many studies and researchers are still trying to achieve more durable structures.

With more recent developments in nanotechnology, cement composites performance is being improved with the utilization of nanomaterial as an engineering material [3,4]. Carbon nanotubes (CNTs) along with other carbon-based nanomaterials have been used to enhance the mechanical properties of cement composites by controlling the cracks at the nanoscale level [5,6,7]. Musso et al. [5] discovered that compression resistance and the modulus of rupture in plain cement paste is able to produce an improvement of around 10%–20% and 14%–34%, respectively, by adding 0.5% in weight of pristine and annealed multi-wall carbon nanotubes (MWCNTs). Konsta-Gdoutos et al. [6] also noted that adding 0.08% weight of cement CNTs to cement paste is able to increase the flexural strength by 25% in comparison to plan cement paste. Cwirzen et al. found that the addition of CNTs into plain cement paste is able to increase the compressive strength by about 50% [7].

Graphene oxide (GO) is currently attracting interest because of its unique properties that enable it to effectively improve the properties of cement-based materials. Researchers have revealed that GO with oxygen-containing functional groups is capable of improving the performance of cement composites [8,9,10,11,12,13]. Lv et al. [8] investigated the effects of GO nanosheets on the shapes and formation process of cement hydration crystals. They also observed that adding low dosages of GO (<0.03%) forms flower-like crystals and polyhedral or lamellar crystals at high dosages (>0.03%). Moreover, it was noted that compressive strength increased 34.3% and 38.1% by adding 0.03% and 0.05% of cement weight GO to plain cement paste, respectively. The flexural strength showed higher improvement and increased by 52.4% and 52.3%, respectively. Wang et al. [9] reported that incorporating 0.05% weight of GO into cement paste is able to increase the 28th-day compressive and flexural strength by 40.4% and 90.5%, respectively; the corresponding increases of 24.4% and 70.5% for hardened mortar. An improvement in compressive strength between 15% and 33% and flexural strength between 41% and 59% over ordinary Portland cement paste was reported after the addition of 0.05% weight GO [10]. Investigation into the effects of graphene oxide nanoplates (GONPs) revealing the properties of cementitious materials was accomplished by Tong et al. [11]. They found that GONPs reshaped the microstructure of cement paste and showed better interfacial bonds between GONPs and C–S–H gels. The compressive strength of mortar samples also improved because of the role of functional groups of graphene oxide. Gong et al. worked to investigate the effect of GO on Portland cement paste and found that adding 0.03% by weight GO sheets to the plain cement paste was able to enhance the tensile and compressive strength by more than 40%. However, a reduction in workability was observed [12]. Lv et al. [13] revealed that the compressive, flexural, and tensile strength of cement paste increased significantly by around 40%, 60%, and 79%, respectively, when 0.03% weight GO was added.

It has been hypothesized that the carbon-based material is able to improve concrete permeability by improving the pore structure, which results in improved resistance to fluid ingress and chemical attacks. There is a direct relationship that exists between concrete durability and the mobility of fluids with concrete [1]. The durability is related to the ease with which liquids and gasses are able to enter the concrete [2,14], which are denied as transport properties. Transport properties highly depend on pore size distribution, total porosity, as well as pore connectivity and its tortuosity [15,16]. Recently, it was recognized that sorptivity is a significant index of concrete durability [17]. The durability of concrete can be improved if the resistance to water penetration is increased [18]. It is vital to analyze the porosity of nano-reinforced cement because of the close relation to the mechanical properties. Many techniques can be utilized to quantify the porous nature of cement such as water sorptivity, nanoindentation, and mercury intrusion porosimetry (MIP). Nanomaterials play an instrumental role in refining the pore structure. For instance, CNTs are able to densify the cement microstructure by filling the pores between hydration products of cement. CNTs also reduce the porosity of the cement composite by reducing the number of mesopores, which are pores less than 50 nm in diameter [19]. Nochaiya et al. [20] revealed that the total porosity and total surface area diminish as MWCNT content is increased by up to 1.0% of the cement weight. The cement composites microstructure and transport properties (i.e., water sorptivity and water permeability) are improved with the addition of a small amount of MWCNTs to the composite as revealed by Han et al. [21]. Kim et al. [22] utilized 0.15% by cement weight of CNTs dispersed with 10% silica fume and concluded that compressive strength is affected by total porosity and by CNT dispersion. GO has been shown to have a profound impact on the pore structure and surface area at the nanoscale. The increased surface area directly corresponds to the development of a highly porous phase. Small pores, measuring between 1 and 10 nm (also called gel pores), are made up of a pore system in C–S–H gel. The porosity of GO-cement described in a pore size distribution was characterized by using an alternative method, which was in fact MIP [12]. The inclusion of GO was able to successfully refine the microstructure of cement composite by lowering the number of capillary pores (between 10 nm and 10 µm) by 27.7%, and this is related to the accelerated hydration owing to the 2D shape of GO.

Previous studies focused on the effect of GO sheets, prepared with common chemical processes (e.g., Hummer’s method) to produce GO sheets with full oxidations (edges, top, and bottom). These methods make the price of GO very high (approximately $100/g), which is one of the major challenges in promoting GOs in large-scale constructions. The high price of this GO limits its practical usage within the construction industry. The graphene oxide used in this research is produced by a ball milling process along with common reactants. Graphite powder was subjected to milling with non-toxic oxidizing agents. The result was edge-oxidized graphene oxide (EOGO) with a few layers. The direct milling process could achieve a dramatic reduction in cost in the manufacture of EOGO by eliminating hazardous waste disposal [23]. Therefore, this innovative mechanochemical process can reduce the price of graphene oxide to under $1.0/g. Consequently, this low cost alternative nanomaterial can be used in construction fields due to the economic advantages. In addition, EOGO can be used to improve the electrical and thermal conductivity of polymers, coating, and composites [24].

The other challenge to introduce GO into large scale constructions is the dispersion method of GO. Using a sonication method to disperse GO in water is well known as the ideal method [4,25]. However, this method may not be practical for high quantities of cement paste or concrete. Therefore, in this study, EOGO is dispersed as powder in cement before mixing with water to investigate the feasibility of using EOGO as an additive material. The interaction of cement particles with the conventional GO will be higher than EOGO because the oxygen-containing functional groups in GO are higher compared to those in EOGO. This may cause higher agglomeration of cement particles with GO when compared with EOGO. The Van der Walls force between GO layers can be weakened with the use of the edge of the oxygen-containing functional groups [26,27], and this will assist in giving a better dispersion of EOGO as powder in cement composites compared to GO. Therefore, EOGO and its mixing methods should be carefully examined to observe the effects that can be had on cement composite properties along with the feasibility of EOGO as an additive material.

In this paper, two mix design methods are used: (1) Dry-mix design, where EOGO and cement powders are mixed before cement paste and mortar formation and (2) Wet-mix design where a sonicator is used for 60 minutes to disperse EOGO into water while using that as the mixing water for cement paste and mortar mixes. To quantify the difference between dry and wet-mix design methods, this research investigates the effect of different mixing methods of EOGO in the cement paste and mortar, including mechanical properties, total porosity, and sorptivity. Five percentages of EOGO between 0.01% and 1.0% by cement weight for both mix design methods were used in this study.

## 2. Materials and Methods 

### 2.1. Materials

#### 2.1.1. Cement and Fine Aggregate

**Figure 1:**
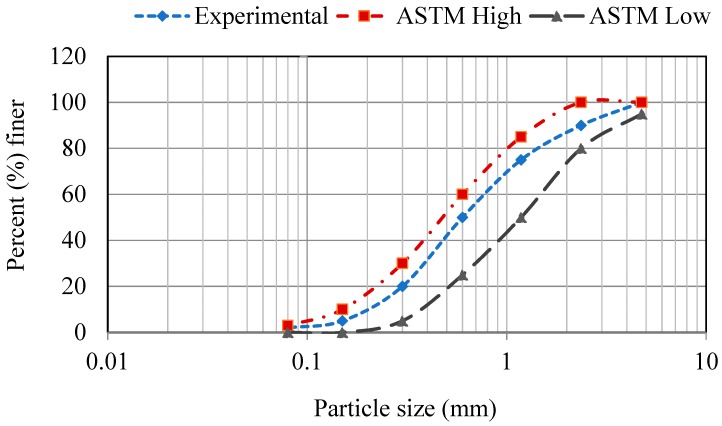
Gradation curves for fine aggregate and ASTM C33 (American Society for Testing and Materials) grading requirements.

Ordinary Portland cement (OPC) type I according to ASTM C150 [28] (American Society for Testing and Materials) was used in this research. The chemical compositions of the OPC can be found in Table 1. Fine aggregate (sand) used for the mortar samples was obtained from CEMEX (Orlando, FL, USA) with a gradation per ASTM C33 [29] as shown in Figure 1.

#### 2.1.2. Edge-Oxidized Graphene Oxide (EOGO)

**Figure 2:**
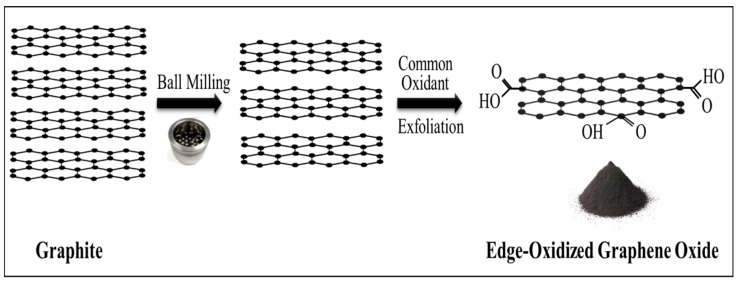
Production methods for producing edge-oxidized graphene oxide (EOGO).

Edge-oxidized graphene oxide was supplied by Garmor, Inc. (Orlando, FL, USA) [24]. The production process of EOGO used in this research is different from the common chemical processes to produce GO such as Hummers’ method [30]. EOGO is produced by a ball milling process of graphite powder with an oxidizing agent in a controlled environment such as optimum shearing and minimized collision forces, whereas the production of GO depends on a chemical process involving strong agents and acids such as KMnO_4_, H_2_SO_4,_ and H_2_O_2_ [30]. The ball milling process oxidizes the graphite along the edges while the milling media simultaneously exfoliates the layers in a zipper-like fashion. This process yields edge-oxidized graphene oxide that is composed of a few layers of graphene. In addition, Figure 2 shows the manufacturing process of ball-milled EOGO.

**Figure 3:**
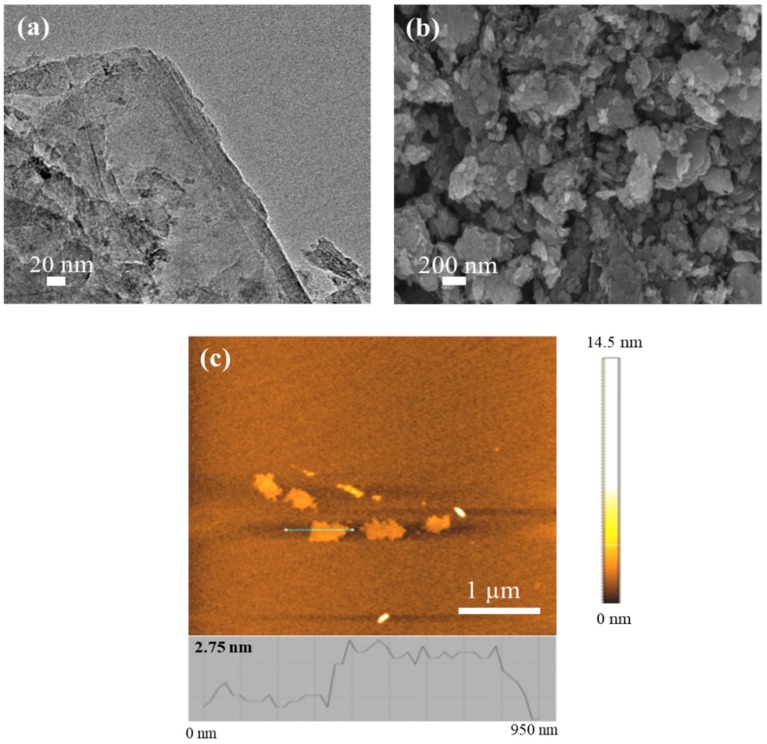
(**a**) Transmission electron microscopy (TEM) image of EOGO, (**b**) scanning electron microscopy (SEM) image of EOGO, and (**c**) atomic force microscopy (AFM) image and height profile of EOGO [24].

EOGO was characterized by Transmission Electron Microscopy (TEM), Scanning Electron Microscopy (SEM), and Atomic Force Microscopy (AFM). The results of TEM, SEM, and AFM for EOGO are illustrated in Figure 3. The TEM image (Figure 3a) clearly shows the multi-layer structures with overlapping areas of EOGO. Figure 3b shows the SEM image of EOGO, and it can be observed that EOGO is a wrinkled layer and it is possible to distinguish the rough surface due to the mechanical process (ball-milling). Figure 3c shows the AFM image of EOGO and illustrates that EOGO flakes have irregular shapes with a dimension of about 400 nm to 500 nm and a thickness of about 2.75 nm, which proves that EOGO has multi-layer structures.

The EOGO content as a percentage of the cement weight was added into cement paste and mortar. For instance, 0.01% of EOGO means 0.01% by cement weight of EOGO. The chemical compositions and physical properties of EOGO are summarized in Table 2.

### 2.2. Preparation of Cement Paste and Mortar Specimens

The cement paste and mortar with EOGO were prepared at different contents of EOGO, varying from 0.01% to 1.0% by weight of cement. Two different mix designs, which are dry and wet-mix designs, were explored in order to identify the best performing mix design for both cement paste and mortar. The dry-mix design of cement paste was prepared according to ASTM C305-14 [31] as the following steps: (1) EOGO powder was manually mixed with cement for a minute prior to applying shearing force. (2) The mixing water was poured into the EOGO-cement mixture and held for 30 s. (3) The mixture was mixed by using a shear mixer for 30 s. (4) The mixer was stopped for 15 s. (5) The mixing was finished by mixing the paste for a minute. On the other hand, the wet-mix design of cement paste involves the following steps: (1) To disperse EOGO in water for the wet-mix design, EOGO powder was first poured into water. (2) A sonicator was utilized for 60 min in order to disperse the EOGO into the water and this solution was then used as the mixing water. (4) Steps 2 to 5 of the dry-mix design procedures were repeated for mixing.

The dry-mix design of mortar was prepared by the following steps: (1) EOGO powder was manually mixed with cement for a minute. (2) The mixing water was poured into EOGO-cement mixture. (3) The mixture was mixed for 30 s. (4) All the quantity of sand was slowly added over a 30s period while mixing. (5) The mixing was continued for another 30 s after adding the sand. (6) The mixer was stopped for 90 s. (7) The mixing was finished by mixing the mortar for a minute. The wet-mix design procedures of mortar involved the following steps: (1) EOGO powder was poured into the water and sonicated for 60 min. (2) The cement was added to the EOGO solution. (3) Steps 3 to 7 of the dry-mix design of mortar were repeated to complete the mixing.

The weight ratio of water to cement was kept at 0.5 for cement pastes and 0.45 for mortars. Mix proportions of cement pastes and mortars can be found in Table 3. The control cement paste and mortar samples are denoted as CP and CM, respectively. The EOGO-cement paste specimens are named as GPD for cement paste mixed with dry-mix design and GPW for cement paste mixed with wet-mix design. Similarly, The EOGO-mortar specimens are named GMD for dry-mix design and GMW for wet-mix design. The number after the abbreviation represents the percentage of EOGO by weight of cement.

### 2.3. Test Methods

#### 2.3.1. Compressive and Flexural Strength Tests

To evaluate the influence of EOGO on the mechanical properties, a compressive strength test was conducted on cubic specimens (50 mm × 50 mm × 50 mm) of EOGO-cement composites according to ASTM C109 [32]. The flexural strength test was performed on 50 mm × 100 mm × 25 mm prisms according to ASTM C348 [33]. A total of 264 specimens were prepared for these tests. All specimens were cured at 20 °C and 90% relative humidity and tested at 7 and 28 days. All tests were conducted in triplicate to evaluate the influence of EOGO content on the strengths of cement composites, and the results were averaged.

#### 2.3.2. Porosity Test 

Effect of the mixing method of EOGO on the total porosity of EOGO-cement pastes and mortars was investigated by conducting the porosity test according to ASTM C1754/C1754M-12 [34] which is a gravimetric method. Two cylinders with 75 mm diameter and 150 mm height were prepared for each mix. A total of 88 specimens were prepared for this test. The test was performed at 7 and 28 days. At first, the submerged mass of each specimen was measured until the submerged mass became constant. Secondly, the specimens were placed in an oven at a temperature of 38 ± 5 °C and the dried mass of the specimens were measured every 24 h until the mass became constant.

#### 2.3.3. Water Sorptivity Test

**Figure 4:**
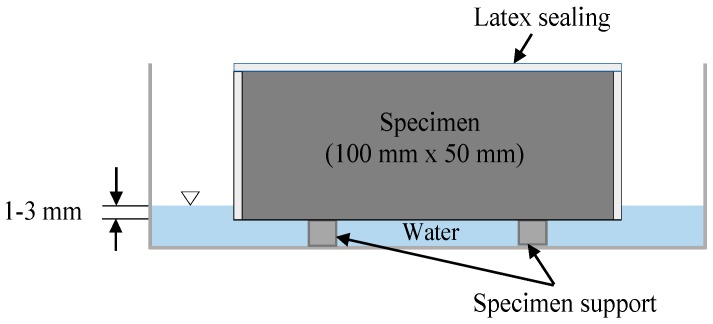
Schematic of sorptivity test setup.

The water absorption rate of cement composites was determined by conducting a water sorptivity test for each cement paste and mortar specimen in accordance with ASTM C1585-13 [35]. Two-disc specimens (100 mm in diameter and 50 mm in height) were prepared for each mix. A total of 44 specimens were prepared for this test, and it was performed at 28 days. The water absorption rate was measured by exposing the bottom surface of cement paste and mortar specimens to water. The other surfaces were sealed with latex based water proof paint. As illustrated in Figure 4, the water level in the test setup was maintained approximately 2 mm from the bottom of the specimens as specified in the standard method. The weight variation of each specimen was recorded at specific time intervals after the first contact with water. Mainly, this test determines the increase in cement paste or mortar prism masses due to capillary-rise absorption. The mathematical equation to calculate the absorption can be expressed as:

(1) $$I=\frac{m_{t}}{a\times d}\;$$

where *I* = is the absorption (mm), $m_{t}$ is the change in specimen mass (g) at the time *t*, *a* is the exposed area of the specimen (mm^2^), and *d* is the density of the water (g/mm^3^).

## 3. Results

### 3.1. Effect of EOGO on the Mechanical Properties of Cement Paste and Mortar 

**Figure 5:**
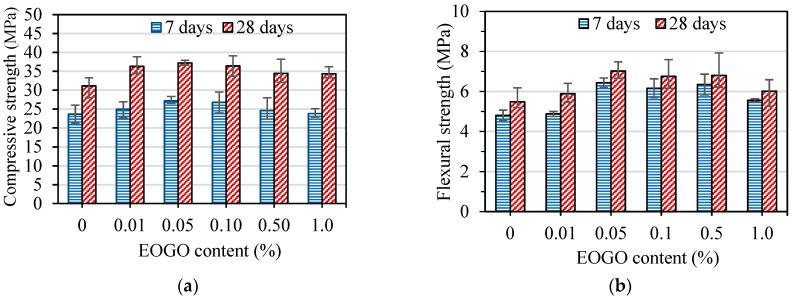
(**a**) Compressive strength and (**b**) flexural strength of EOGO-cement pastes with the dry-mix design method.

The results of the strength tests for cement pastes using the dry-mix design method are shown in Figure 5. The strength increased with increasing EOGO content until it reached 0.05%, followed by a reduction in the strength with a further increase in EOGO content to 1.0%. The compressive and flexural strengths of control specimens (CP) were 23.66 MPa and 4.8 MPa at 7 days, and 31.12 MPa and 5.48 MPa at 28 days, respectively. Figure 5a shows that specimens containing 0.05% and 0.1% of EOGO (GPD 0.05 and GPD 0.1) exhibited 14.93% and 13.11% increase in compressive strength at 7 days and 19.6% and 17% at 28 days, respectively compared with CP. Figure 5b shows that the flexural strength of GPD 0.05 and GPD 0.1 increased by 33.95% and 28.18% at 7 days, and 28.02% and 23.18% at 28 days, respectively compared with those of CP. Therefore, the optimum EOGO content was 0.05% (GPD 0.05). However, the strengths of specimens containing 0.1% (GPD 0.1) were slightly lower than those of GPD 0.05. These strength results indicate that it is feasible and could be effective for the strength improvement of EOGO-cement paste even if there is no ultrasonication for the dispersion of EOGO. In addition, there was a tendency that the error bar of compressive strength results becomes wider in proportion to the addition of EOGO after 0.05% EOGO. This increased uncertainty of strength may be due to a possible agglomeration of EOGOs and reduction of workability in the cement matrix as well. Despite the mixing method with dispersion of EOGOs, it is still a challenge to control the dispersion of EOGO in the cement matrix because of “growth” of cement hydration over time, particularly when a high percentage of EOGO is added. Heterogeneity in cement and sand from the microscale perspective can also cause the variations in strength tests, which is not uncommon in practice. 

**Figure 6:**
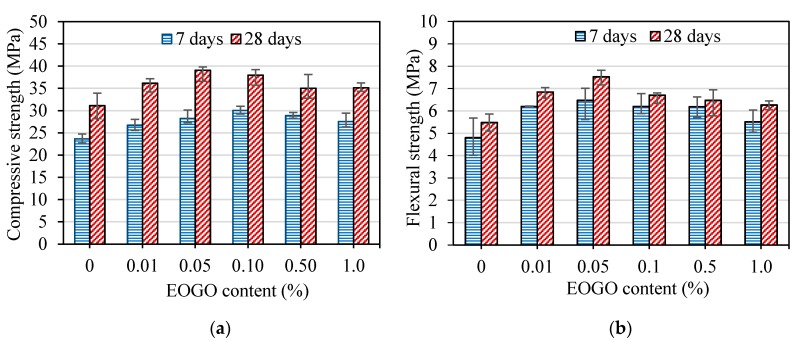
(**a**) Compressive strength and (**b**) flexural strength of EOGO-cement pastes with the wet-mix design method.

To compare the effect of the different mix design methods, namely the dry and wet-mix design methods, specimens mixed by wet-mix design methods (GPW) were tested. The results of the compressive and flexural strength tests of those specimens are shown in Figure 6. The compressive and flexural strengths of GPW specimens are higher than those of CP and show a similar pattern of strengths improvement compared with GPD specimens. Figure 6a shows that the compressive strength of GPW 0.05 and GPW 0.1 increased by 19.39% and 26.92% at 7 days and 25.54% and 16.20% at 28 days, respectively compared with those of CP. Moreover, Figure 6b shows that the flexural strength of GPW 0.05 and GPW 0.1 increased by 34.85% and 29.12% at 7 days and 37.34% and 22.25% at 28 days, respectively compared with those of CP. These results show that GPW 0.05 and GPW 0.1 exhibited a higher improvement of the mechanical properties of GPW specimens among the other percentages.

**Figure 7:**
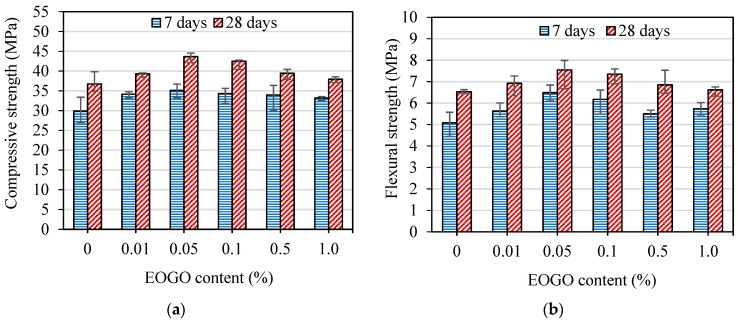
(**a**) Compressive strength and (**b**) flexural strength of EOGO-mortars with the dry-mix design method.

The results of the strength tests for the mortar samples prepared by the dry-mix design are illustrated in Figure 7. The compressive strength of specimens without EOGO (CM) and EOGO-mortar specimens (GMD) are shown in Figure 7a. The compressive strengths of CM were 29.9 MPa and 36.8 MPa at 7 and 28 days, respectively. All GMD specimens showed higher compressive strengths compared with CM. Specimens with EOGO contents of 0.05% (GMD 0.05) and 0.1% (GMD 0.1) exhibited the greatest compressive strength among all specimens. The compressive strength of GMD 0.05 and GMD 0.1 were 35.1 MPa and 34.3 MPa at 7 days, representing approximately 17% and 15% increases compared with CM. The compressive strength of GMD 0.05 and GMD 0.1 were 43.7 MPa and 42.5 MPa at 28 days, representing approximately 19% and 16% increases compared with CM. Figure 7b shows the flexural strength of EOGO-mortar with different EOGO contents. The flexural strengths of CM were 5.1 MPa and 6.9 MPa at 7 and 28 days, respectively. The flexural strength of GMD 0.05 and GMD 0.1 were 6.5 MPa and 6.2 MPa at 7 days, representing 17% and 15% increases compared with CM while the flexural strengths of those specimens were 7.5 MPa and 7.4 MPa after 28 days of curing, representing 19% and 16% compared with CM. The results indicate a significant increase in the mechanical properties of EOGO-mortars when 0.05% and 0.1% of EOGO were incorporated using the dry-mix design.

**Figure 8:**
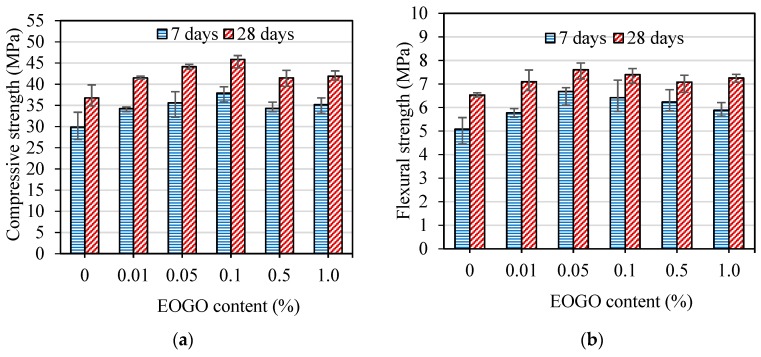
(**a**) Compressive strength and (**b**) flexural strength of EOGO-mortars with the wet-mix design method.

The strength tests were also conducted on mortar specimens prepared by the wet-mix design method to compare the effect of the mix design on the mechanical properties of mortars containing EOGO. Figure 8 shows the compressive and flexural strengths of the EOGO-mortar specimens. Figure 8a illustrates that the compressive strengths of all GMW samples are higher than the CM specimens at both curing times. It can be seen that GMW 0.05 and GMW 0.1 had the greatest compressive strengths compared with other GMW specimens. The compressive strength at 7 days of GMW 0.05 and GMW 0.1 were 35.6 MPa and 37.9 MPa, respectively, which increased 19% and 27% compared with CM. The compressive strength at 28 days of GMW 0.05 and GMW 0.1 were 44.1 MPa and 45.8 MPa, respectively, which increased 20% and 25% compared with CM. Figure 8b shows that the flexural strength of GMW 0.05 and GMW 0.1 were 6.7 MPa and 6.4 MPa, respectively at 7 days, which are 31% and 26% higher that of CM. The flexural strengths after 28 days curing of GMW 0.05 and GMW 0.1 were 7.6 MPa and 7.4 MPa, respectively, which are 16% and 13% higher that of CM.

**Figure 9:**
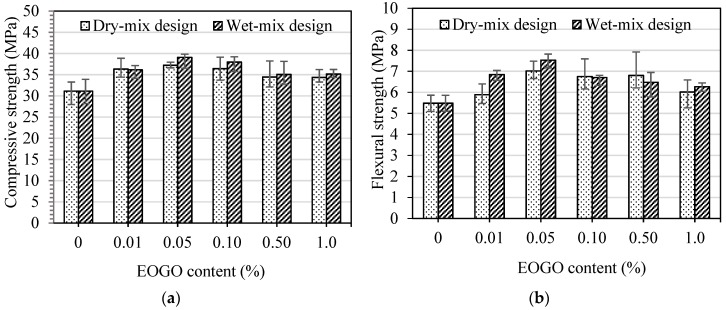
Comparison between the dry and wet-mix design methods of EOGO-cement pastes: (**a**) Compressive strength at 28 days and (**b**) flexural strength at 28 days.

**Figure 10:**
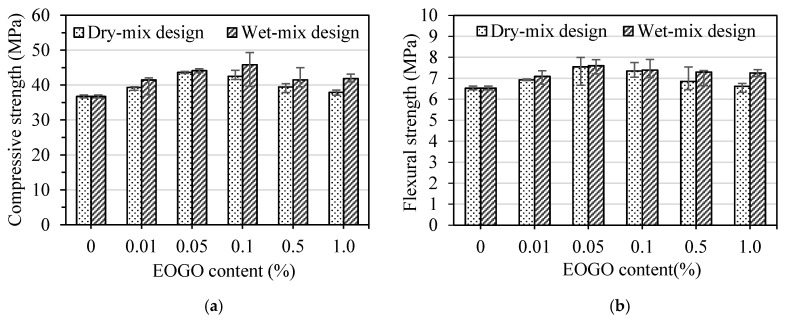
Comparison between the dry and wet-mix design methods of EOGO-mortars: (**a**) Compressive strength at 28 days and (**b**) flexural strength at 28 days.

On comparison of the dry and wet-mix designs, the wet-mix method is shown to exhibit slightly higher compressive and flexural strengths at 28 days over all percentages of EOGO for cement paste as illustrated in Figure 9. For example, the compressive and flexural strengths at 28 days of GPW 0.05 are around 5% and 7%, respectively higher than GPD 0.05 as shown in Figure 9a,b. Figure 10a,b show that the strengths of GMW specimens are somewhat similar to those of GMD specimens when the EOGO contents were 0.1% and lower. However, when the EOGO contents in mortars were 0.5% and higher, the compressive and flexural strengths of GMW specimens were higher than those of GMD. For instance, the compressive and flexural strengths at 28 days of GMW 1.0 are 10.5% and 10.6% higher than those of GMD 1.0, respectively. This is due to the fact that the large content of EOGO with the wet-mix design (GMW 1.0) has a better exfoliation and dispersion compared to GMD 1.0 with the dry-mix design. Consequently, GMW 1.0 has a better effect on the mechanical properties of EOGO-mortars than GMD 1.0.

### 3.2. Effect of EOGO on the Porosity of Cement Paste and Mortar Specimens

**Figure 11:**
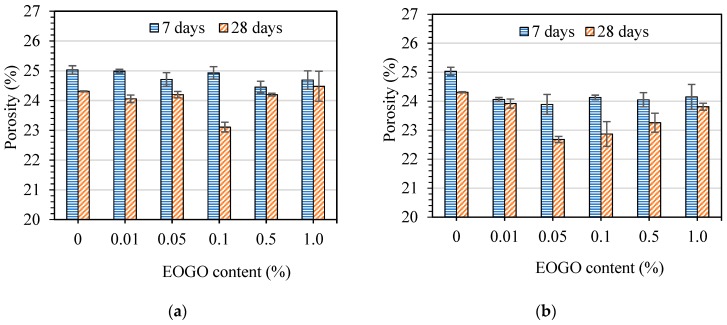
Effect of EOGO on the porosity of cement pastes: (**a**) Dry-mix design and (**b**) wet-mix design.

Figure 11a shows the relationship between the porosity of the control mix and the mixes with different EOGO contents cured at 7 and 28 days utilizing the dry-mix design method. In addition, it can be clearly seen that the porosity of the GPD samples at 7 days slightly decreased when the EOGO contents were 0.05% and more when compared to the control cement paste (CP). The porosity of all GPD specimens cured at 28 days was less than the CP. This indicates that the addition of EOGO using the dry-mix design reduces the porosity of EOGO-cement paste specimens. However, the reduction in the porosity of EOGO-cement pastes at 7 and 28 days was not significant. For instance, the greatest reduction in the porosity cured at 28 days was around 5% when the EOGO content was 0.1%.

To compare the effect of different mix design methods on the porosity of cement paste, the porosity of GPW samples prepared by using the wet-mix design were measured. Figure 11b illustrates the effect of the addition of EOGO on the porosity of cement paste cured at 7 and 28 days. It can be observed that GPW 0.05 exhibits the highest reduction in the porosity among the GPW samples at 7 and 28 days. The porosity of GPW 0.05 cured at 7 and 28 days decreased by around 4.5% and 6%, respectively compared to CP. In addition, it can be seen that the porosities at 7 and 28 days of GPW samples are less than that of GPD samples. For example, the porosity at 28 days of GPW 0.05 is around 6% lower than that of GPD 0.05. 

**Figure 12:**
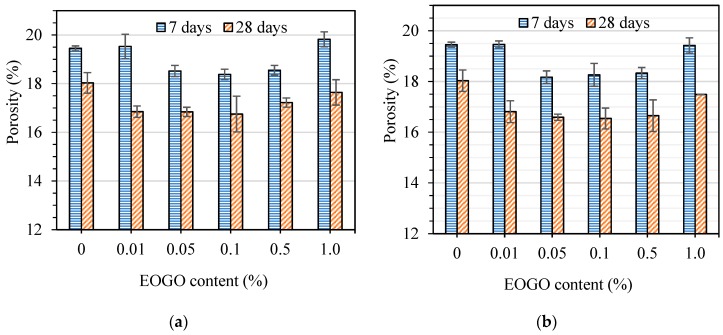
Effect of EOGO on the porosity of mortars: (**a**) Dry-mix design and (**b**) wet-mix design.

To investigate the effect of different percentages of EOGO and different mix design methods on the total porosity of cement mortar, the porosity was measured for both dry and wet-mix design methods. Figure 12a shows the effect of the addition of different contents of EOGO on the porosity of mortar mixes cured at 7 and 28 days using the dry-mix design. The results indicate that the total porosity of GMD specimens decreased compared to the control mortar (CM) specimen. It can be seen that the porosity decreases when the EOGO content increases up to 0.1%, then it begins to increase when 0.5% and more of EOGO is added. The porosity at 28 days of GMD 0.05 and GMD 0.1 has the most reduction in the total porosity among all GMD samples which decreased by 6.6% and 7.1% compared to CM. This result demonstrates that using the dry-mix design can affect the total porosity of EOGO-cement mortars when adding moderate percentages of EOGO (0.05% and 0.1%).

To compare the effect of different mix design methods on the total porosity of cement mortar, the porosity cured at 7 and 28 days of GMW mixes that were prepared by using the wet-mix design were measured. Figure 12b shows the effect of various EOGO percentages on the porosity of cement mortars using the wet-mix design method. The results show that all GMW samples are lower than the CM. The general trend of the results is similar to the results found when the dry-mix design was used. It is clearly seen that GMW 0.05 and GMW 0.1 have the highest reduction in porosity for both curing times. The porosity at 28 days of GMW 0.05 and GMW 0.1 decreased by approximately 7.9% and 8.2% compared to the CM. 

### 3.3. Effect of EOGO on Water Sorptivity of Cement Paste and Mortar

**Figure 13:**
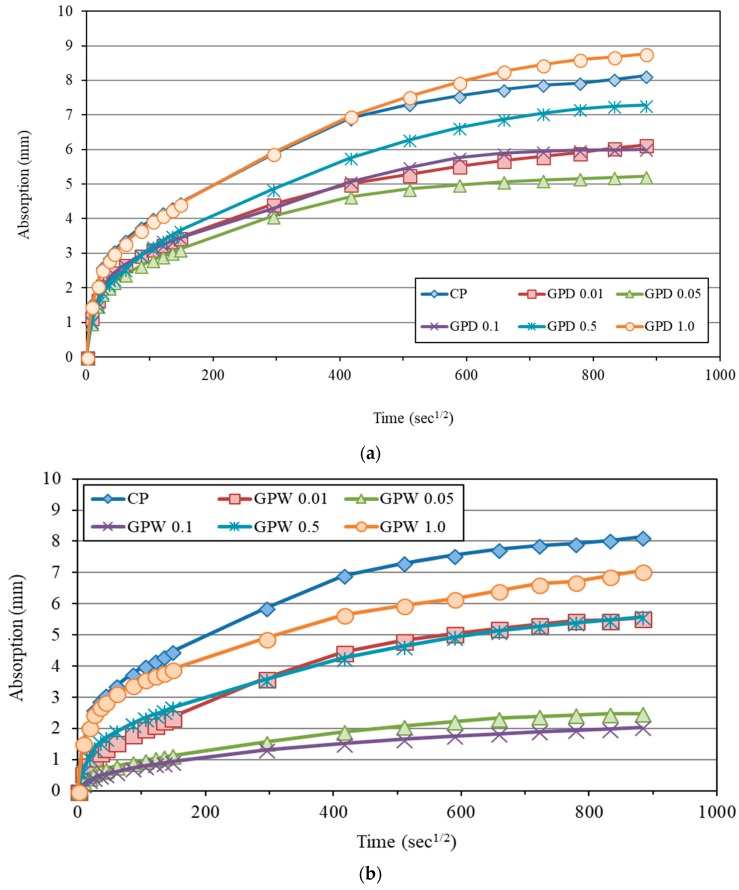
Water sorptivity of EOGO-cement pastes: (**a**) Dry-mix design and (**b**) wet-mix design.

The water sorptivity of EOGO-cement composites were measured over nine days. Sorptivity is recognized as a significant index of concrete durability. Figure 13 shows the results of water sorptivity for control mix (CP) and EOGO-cement paste (GPD) mixes cured at 28 days. Figure 13a,b describes the water sorptivities of EOGO-cement pastes made of dry and wet-mix design methods, respectively. The graphs in Figure 13 plot the water absorption responses on the y-axis against the square root of time on the x-axis according to ASTM C1585-13 [35]. The water absorption process can be divided into two main phases, which are an initial sorptivity and a secondary sorptivity. The initial sorptivity is a linear regression measured up to the first 6 hours and the second sorptivity is a linear regression measured from day 1 to day 9. The addition of EOGO in the cement matrix using the dry-mix design was found to be effective in reducing the water suction mainly in GPD 0.01, GPD 0.05, and GPD 0.1 samples as shown in Figure 13a. The results of the initial and secondary sorptivity are presented in Table 4. The sorptivity of GPD 0.01, GPD 0.05, and GPD 0.1 were reduced by 19.7%, 29%, and 23.5%, respectively for the initial sorptivity and 20%, 48.6%, and 22.9%, respectively for the second sorptivity compared to CP. In addition, the results show that adding 0.5% and 1.0% of EOGO to cement paste mix (GPD 0.5 and GPD 1.0) has no or negative effect on the initial and secondary sorptivity. This agrees with the porosity test which illustrates higher porosity of GPD 0.5% and 1.0% at 28 days among the other percentages.

To compare the effect of the mixing method of EOGO-cement pastes on the water absorption rate, the water sorptivity test was performed on EOGO-cement paste mixes using the wet-mix design method. Figure 13b shows the water absorption and the square root of time curves for CP and GPW mixes cured at 28 days. The results show that the water absorption rates of all GPW specimens are less than that of CP. The results are shown in Table 4. The water absorption rate of GPW 0.05 and GPW 0.1 was significantly decreased compared to the other mixes. Compared to CP, the initial sorptivity of GPW 0.05 and GPW 0.1 samples was reduced by 70.5% and 73.8%, respectively while the secondary sorptivity was reduced by 57.1% and 65.7%, respectively. This is consistent with the porosity test at 28 days which shows low porosity of GPW 0.05 and GPW 0.1 compared to the other mixes. Similar to the dry-mix design, adding 0.5% and 1.0% of EOGO to matrix (GPW 0.5 and GPW 1.0) shows lower effect on initial and secondary sorptivity of EOGO-cement paste compared to GPW 0.05 and GPW 0.1. This indicates that the increase of EOGO content beyond 0.1% would not further decrease the water absorption rate due to the agglomeration effect. It can be observed that the water absorption rates (initial and secondary sorptivity) of GPW specimens are lower than those of GPD specimens. For example, the initial sorptivity of GPW 0.05 and GPW 0.1 are approximately 58% and 65% lower than GPD 0.05 and GPD 0.1. Furthermore, it is noticeable that using the wet-mix design has a better effect than the dry-mix design when the EOGO percentage is increased to 0.5% and 1.0%, and this is referred to by better dispersion of EOGO with the wet-mix design compared to the dry-mix design, leading to a reduction in the agglomeration of a large amount of EOGO, reducing the water sorptivity of the matrix.

**Figure 14:**
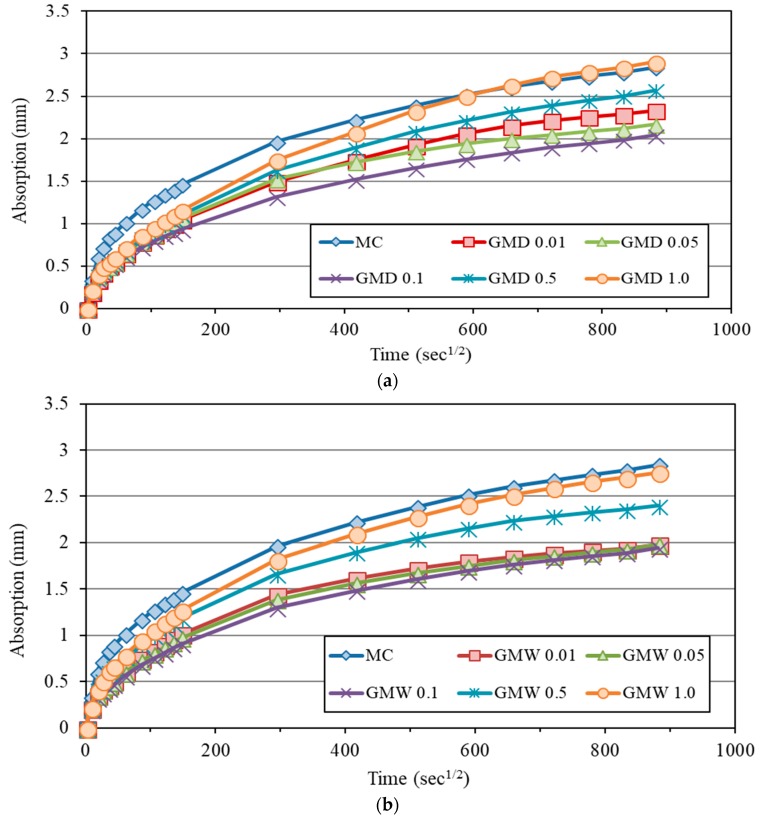
Water sorptivity of EOGO-mortars: (**a**) Dry-mix design and (**b**) wet-mix design.

To investigate the effect of different percentages of EOGO and different mix design methods on the water absorption rate of cement mortar, the water sorptivity test was conducted for both dry and wet-mix design methods. Figure 14a,b shows the cumulative absorption of different EOGO-mortar mixes using the dry and wet-mix designs, respectively. Figure 14a shows that the addition of EOGO to mortar using the dry-mix design reduces the water absorption of EOGO-mortar specimens. The results are summarized in Table 5. It can be seen that the GMD 0.05 and GMD 0.1 exhibit the lowest initial and secondary sorptivity. The same trend was found for the dry-mix design of EOGO-cement paste specimens. The initial sorptivity of GMD 0.05% and GMD 0.1% reduced by 27.3% and 48%, respectively, while the secondary sorptivity of the same samples reduced by 40% and 27.3%, respectively compared to the control sample (CM). This pattern is consistent with the porosity test at 28 days of mortars with the dry-mix design method. The results indicate a less effective addition of EOGO in cement mortar mixes using the dry-mix design when the EOGO percentage is 0.5% or more. The water sorptivity test also was performed on EOGO-mortar specimens using the wet-mix design to compare the effect of the mixing method of EOGO in cement mortars on the water absorption of those specimens. Figure 14b illustrates the plot of water absorption against the square root of time of EOGO-mortar specimens mixed by using the wet-mix design method. It is apparent that the water absorption decreases with the addition of EOGO in cement mortars. GMW 0.05, and GMW 0.1 samples show a significant reduction in the initial and secondary sorptivity. The initial sorptivity of GMW 0.05% and GMW 0.1% was reduced by 48.9.3% and 37.3%, respectively, while the secondary sorptivity of the same samples was reduced by 55.6% and 40%, respectively compared to CM. It is interesting to note that when EOGO contents were 0.5% and 1.0% (GMW 0.5 and GMW 1.0), there was an insignificant effect on the water absorption rate compared to the other percentages of EOGO. This result was observed with both dry and wet-mix design methods for cement paste and with dry-mix design for mortar. In addition, the results of water sorptivity of GMW specimens were slightly lower than those of GMD specimens. For instance, the secondary sorptivity of GMW 0.05 and GMW 0.1 are 10% and 9% lower than GMD 0.05 and GMD 0.1 specimens. This indicates that the wet-mix design has a slightly better influence on water sorptivity of EOGO-mortar mixes than the dry-mix design.

### 3.4. Scanning Electronic Microscopy (SEM) and Energy Dispersive Spectroscopy (EDS) Analysis

**Figure 15:**
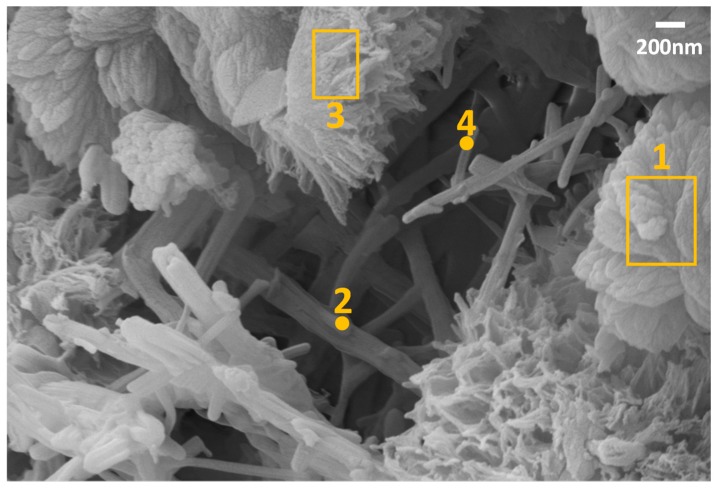
SEM analysis of EOGO nanoflakes-cement composite (0.05% of EOGO) sample with different resolutions.

SEM was conducted to investigate the morphology of EOGO-cement paste. Figure 15 shows a SEM image of the morphology of the hardened cement paste with 0.05% of EOGO. The EOGO-cement paste with 0.05% was chosen because 0.05% was found to be the optimal content for strength improvement. The SEM image shows several needle-shaped ettringite crystals and early age morphologies of amorphous C–S–H.

The locations of EOGO were verified by performing EDS at different spots that are shown in the SEM image. The EDS analysis results are presented in Table 6. The results of the spot 2 and area shots 1 and 3 show high percentages of oxygen and the presence of carbon, while spot 4 shows ettringite which is a byproduct of cement hydration with no presence of carbon element. Moreover, areas 1 and 3 indicate either amorphous of C–S–H or crystalline C–S–H (Jennite). The identification of EOGO in C–S–H, which is the main hydration product responsible for the cement paste strength, indicates that EOGO may act as a seeding material for C–S–H.

## 4. Discussion

The results of the mechanical properties of EOGO-cement composites with dry and wet-mix design methods show significant improvements compared to control cement composites. Previous studies conclude that a small amount of GO provides significant improvements in the mechanical properties of cement composites [13,36,37,38,39,40]. Most of the previous studies used Hummers-produced GO for GO-cement composites. Also, the optimum GO content for the strength improvement ranges from 0.01% to 0.05%. The mechanical properties of EOGO-cement composites exhibited the greatest improvements when EOGO contents were 0.05% and 0.1%. There are two possible reasons why the EOGO optimum content is higher than the optimum GO content. First, the total amount of oxygen groups for EOGO are lower than for GO. EOGO has most of the oxygen groups along the edges while GO has oxygen groups over the entire surface. Second, GO is a single layer of graphite with oxygen groups. On the other hand, EOGO is composed of several layers of graphene. Despite these demerits of EOGO in comparison with GO, from a practical application perspective, EOGO is actually worthwhile to use. The innovative mechanochemical process which produces EOGO can reduce the price of graphene oxide nanoflakes to under $1.0/g; therefore, the use of EOGO in large scale production of concrete becomes applicable.

The EOGO functionality has an impact on the crystal seed growth of the calcium silica hydrate (C–S–H) gels and other hydration products similar to GO [13,41,42,43,44]. Oxygen groups along the edges of the EOGO flakes are able to provide nucleation sites through coordinating with the calcium ions and improving the crystal growth. Thus, EOGO-cement composites were able to achieve higher mechanical properties. In addition, for both mix designs, the significant improvement in mechanical properties on incorporating 0.05% and 0.1% of EOGO can be explained by the significant reduction in capillary pores when the same percentages are added to cement paste and mortar.

The total porosity of cement composites containing EOGO for both dry and wet-mix design methods cured at 28 days are lower than those cured at 7 days. This is because the increase of curing time provides a more suitable environment for cement hydration, reducing the total porosity of the cement based materials [45,46]. A slight reduction in the total porosity of cement composite specimens was noticed after the incorporation of EOGO for both mix designs compared to the control samples. A similar result was reported in cement composites incorporating graphene oxide [12,47]. The possible mechanism is that the EOGO as a nano-scale material can fill the nano and micro-pores of the cement matrix. Additionally, the addition of the 2D shape of graphene oxide accelerates the hydration of cement composites, refining the microstructure by reducing the total porosity [4]. It is interesting to note that increasing the EOGO content to 0.5% and more has less effect in reducing the porosity of the cement composites for both mix designs. This could be because of poor workability caused by the high surface area of EOGO that introduces large pores [47]. 

The results of the water sorptivity tests for both dry and wet-mix design show a significant effect of EOGO on the water sorptivity of cement composites. The water absorption rate is mostly influenced by the capillary pores. The capillary pore size can be classified as: large capillaries or macropores (50–10,000 nm), large mesopores (10–50 nm), and small mesopores (2.5–10 nm) [47,48]. According to Li et al. [47], the addition of graphene oxide has no significant effect on pores larger than 50 µm (50,000 nm), but it significantly reduces the large capillary pores (50–10,000 nm) and can refine the pore structure. Therefore, the pore structure of EOGO-cement composite can be changed but the total porosity of EOGO-cement composite may not be changed much compared to the control specimen. It was found that there is no proportional relation between the water absorption rate and the total porosity of EOGO-cement composite. In other words, the total porosity of cement pastes and mortars was not significantly affected by the addition of EOGO. However, a significant reduction in large capillary pores was detected based on the results of the water sorptivity test. This finding agrees with relevant research results [1,12,47]. One of the possible reasons for the reduction in the water sorptivity is due to the seeding and filling effects on EOGO incorporation in the cement matrix. The second reason for this phenomenon can be attributed to the fact that EOGO refines the capillary pore system and increases the tortuosity of cement composites and this leads to improved resistance of cement composites to water absorption. The large capillary pores of cement composites showed a significant reduction when 0.05% and 0.1% of EOGO were incorporated on the basis of the water sorptivity result. The results also show that further addition of EOGO beyond 0.1% did not further reduce the water absorption rate. This phenomenon may be explained by the introduction of larger pores due to the poor workability of cement composites with 0.5% and more of EOGO [47]. In addition, incorporating 0.5% or more of EOGO would cause agglomeration of EOGO into porous clusters and this leads to more pathways for water to be sucked into by the specimens.

On comparison of the dry and wet-mix designs, the results show that the wet-mix design has a better effect on the compressive and flexural strengths, total porosity, and water absorption rate of cement composites compared to the dry-mix design. This is attributed to the better exfoliation and dispersion of EOGO in the cement matrix with the wet-mix design. The well-dispersed EOGO in the cement matrix may increase the filling and interlocking effect of EOGO, inducing a structure with less pores. Moreover, the well-dispersed EOGO flakes in water with the wet-mix design have a larger surface area compared to the dry powder of the EOGO flakes in the dry-mix design because of lower agglomeration. The larger surface area of EOGO flakes absorbs more free water in the mixes and has more oxygen-containing functional groups, which equates to more nucleation sites. These groups can act as a binder between graphene oxide nanoflakes, and cement paste to obtain greater uniformity of the cement matrix [49,50,51], leading to lower capillary pores and higher strengths.

However, the compressive and flexural strengths gained by the dry-mix design method are sufficiently high as structural materials. In addition, using the dry-mix design showed significant improvements in reducing the capillary pores of cement composites. The use of the edge for the oxygen-containing functional groups is able to make the Van der Walls force between the EOGO layers weaker. This results in good dispersion of the dry powder of EOGO in cement matrix [26,27]. Moreover, by applying the dry-mix design method, the use of EOGO in large scale production of concrete becomes applicable. The study results support the fact that the dry-mix design is economical, feasible, and practical for EOGO-cement composites and can be implemented in the concrete industry.

## 5. Conclusions

This study investigated the effect of different mix design methods of EOGO-cement composites on the mechanical properties, total porosity, and water sorptivity of EOGO-cement composites. Different contents of EOGO ranging from 0.01% to 1.0% were used as an additive in cement paste and mortar. The following conclusions were made on the research findings:

(1) The mechanical properties of all EOGO-cement composites were improved over the control cement composite specimens for both dry and wet-mix designs. It was observed that EOGO-cement composite with 0.05% exhibited the highest strengths at 7 and 28 days. EOGO-composites prepared by the dry- and wet-mix designs show a slight difference in strength.
(2) For both dry and wet-mix designs, the addition of EOGO has a slight effect on the total porosity of cement composites. However, the addition of EOGO in cement composites can significantly refine the pore structure of cement composites according to the sorptivity results. There is a significant improvement of EOGO-cement composite in sorptivity. These sorptivity results indicate that the addition of EOGO results in the discontinuation of capillary pores in the cement matrix, thus leading to improvement in the durability of the cement composites. It was found that EOGO-cement composite with 0.05% mostly showed the greatest improvement in sorptivity.
(3) The petrographic analyses (SEM/EDS) show that EOGO is mostly found in C–S–H which is the main hydration product and is primarily responsible for the strength of cement composite. This observation indicates that EOGO may bridge the C–S–H groups whereby the strength of the EOGO-cement composite can be improved.
(4) The comparison between dry and wet-mix design methods shows that using the wet-mix design method shows slightly better improvement in the mechanical properties, total porosity, and water sorptivity of cement composites compared with the dry-mix design due to the better exfoliation and dispersion of EOGO flakes in the cement matrix. Nevertheless, the dry-mix design method has also significant effects on the mechanical and sorptivity characteristics of cement composites where the optimum content of EOGO (0.05%) increased the compressive and flexural strengths at 28 days of cement paste by around 20% and 28%, respectively. Both the compressive and flexural strengths at 28 days of mortar increased by around 19%. In addition, the sorptivity of cement paste and mortar decreased by approximately 48% and 40%, respectively. Thus, the dry-mix design is a practical, feasible, and economical method for EOGO-cement composites, and can be implemented in the concrete industry.

## Figures and Tables

**Table 1:** Chemical composition of Ordinary Portland Cement (OPC).

Contents	CaO	SiO_2_	SO_3_	Al_2_O_3_	Fe_2_O_3_	Insoluble Residue	Loss on Ignition
(%)	64.90	21.49	0.70	4.21	3.50	1.10	-

**Table 2:** The chemical composition and physical properties of edge-oxidized graphene oxide (EOGO) [24].

Material	Chemical Composition		Physical Properties		
	Carbon (%)	Oxygen (%)	Density (g/cm^3^)	Surface Area (m^2^/g)	Mean Particle Size (nm)
EOGO	90–96	5–10	~1.0	200–300	400–500

**Table 3:** Cement paste and mortar mix proportion (see above for definition of abbreviations).

Specimen ID	*w*/*c* Ratio	Water (mL)	Cement (g)	Sand (g)	EOGO (g)
CP	0.5	1750	3500	-	-
GPD 0.01	0.5	1750	3500	-	0.35
GPD 0.05	0.5	1750	3500	-	1.75
GPD 0.1	0.5	1750	3500	-	3.5
GPD 0.5	0.5	1750	3500	-	17.5
GPD 1.0	0.5	1750	3500	-	35
GPW 0.01	0.5	1750	3500	-	0.35
GPW0.05	0.5	1750	3500	-	1.75
GPW 0.1	0.5	1750	3500	-	3.5
GPW 0.5	0.5	1750	3500	-	17.5
GPW 1.0	0.5	1750	3500	-	35
CM	0.45	780	1686	4215	-
GMD 0.01	0.45	780	1686	4215	0.1686
GMD 0.05	0.45	780	1686	4215	0.843
GMD 0.1	0.45	780	1686	4215	1.686
GMD 0.5	0.45	780	1686	4215	8.43
GMD 1.0	0.45	780	1686	4215	16.86
GMW 0.01	0.45	780	1686	4215	0.1686
GMW0.05	0.45	780	1686	4215	0.843
GMW 0.1	0.45	780	1686	4215	1.686
GMW 0.5	0.45	780	1686	4215	8.43
GMW 1.0	0.45	780	1686	4215	16.86

**Table 4:** Initial and secondary sorptivity of cement paste mixes for dry and wet-mix design *.

Samples	Initial Sorptivity (mm/s^1/2^)	Reduction Compared to CP (%)	Secondary Sorptivity (mm/s^1/2^)	Difference with CP (%)
CP	0.0183	-	0.0035	-
GPD 0.01	0.0147	−19.7	0.0028	−20.0
GPD 0.05	0.0130	−29.0	0.0018	−48.6
GPD 0.1	0.0140	−23.5	0.0027	−22.9
GPD 0.5	0.0170	−7.1	0.0040	+14.3
GPD 1.0	0.0183	0	0.0042	+20.0
GPW0.01	0.0118	−35.5	0.0030	−14.3
GPW0.05	0.0054	−70.5	0.0015	−57.1
GPW 0.1	0.0048	−73.8	0.0012	−65.7
GPW 0.5	0.0126	−31.1	0.0033	−5.7
GPW 1.0	0.0146	−20.2	0.0034	−2.9

* (−ve means reduction, and +ve means increase compared to CP).

**Table 5:** Initial and secondary sorptivity of mortar mixes for the dry and wet-mix designs *.

Samples	Initial Sorptivity (mm/s^1/2^)	Reduction Compared to CM (%)	Secondary Sorptivity (mm/s^1/2^)	Difference with CM (%)
CM	0.007	-	0.0014	-
GMD 0.01	0.0057	−22.8	0.0013	−7.7
GMD 0.05	0.0055	−27.3	0.001	−40.0
GMD 0.1	0.0047	−48.9	0.0011	−27.3
GMD 0.5	0.0058	−20.7	0.0016	+12.5
GMD 1.0	0.0061	−14.8	0.0019	+26.3
GMW0.01	0.0054	−29.6	0.0011	−27.3
GMW0.05	0.0047	−48.9	0.0009	−55.6
GMW 0.1	0.0051	−37.3	0.001	−40.0
GMW 0.5	0.0062	−12.9	0.0012	−16.7
GMW 1.0	0.0069	−1.4	0.0016	+12.5

* (−ve means reduction, and +ve means increase compared to CM).

**Table 6:** Energy Dispersive Spectroscopy (EDS) analysis on EOGO nanoflake-cement composite.

Shots	C (%)	O (%)	Al (%)	Si (%)	S (%)	Ca (%)	Pd (%)	Au (%)	Total (%)	Probable Compounds
1 (Area)	12.43	30.05	-	3.17	-	50.93	-	3.42	100	C–S–H+EOGO
2 (Point)	5.29	31.98	4.39	6.82	4.63	46.89	-	-		C–S–H+Ettringite+EOGO
3 (Area)	15.56	39.99	0.86	10.15	-	25.74	4.56	3.15		Jannite+EOGO
4 (Point)	-	13.70	2.58	9.23	5.47	69.01	-	-		Ettringite
